# Investigation of the effects of electromagnetic field treatment of hot spring water for scale inhibition using a fibre optic sensor

**DOI:** 10.1038/s41598-019-47088-6

**Published:** 2019-07-24

**Authors:** Takuya Okazaki, Senshin Umeki, Tatsuya Orii, Ryusuke Ikeya, Aya Sakaguchi, Takamichi Yamamoto, Tomoaki Watanabe, Akira Ueda, Hideki Kuramitz

**Affiliations:** 1 Department of Applied Chemistry, School of Science and Technology, Meiji University, 1-1-1, Higashimita, Tama-ku, Kawasaki, Kanagawa 214-8571 Japan; 2 Graduate School of Environmental Studies, Tohoku University, 468-1, Aoba, Aramaki, Aoba-ku, Sendai, Miyagi 980-8572 Japan; 3 Department of Environmental Biology and Chemistry, Graduate School of Science and Engineering for Research, University of Toyama, 3190, Gofuku, Toyama 930-8555 Japan

**Keywords:** Environmental monitoring, Geochemistry

## Abstract

Treatment with an electromagnetic field, one of the potential techniques to inhibit scale deposition from water, has the advantage of not requiring the addition of any chemicals. Field tests using a fibre optic sensor were conducted to evaluate the effect that the treatment of hot spring water in Matsushiro, Japan with an electromagnetic field had on calcium carbonate scale formation. The optical response to scale deposition recorded by the fibre optic sensor decreased as a consequence of the application of an electromagnetic field, and the effectiveness of scale formation inhibition depended on the frequency of the electromagnetic field. This evidence was compared with results from changes in scale mass measured using the quartz crystal microbalance (QCM) method. Mass increases of the scale formed on the quartz crystal surface in hot spring water were inhibited by electromagnetic field treatment. These results were verified performing a column flow test, whereby the flow rate of hot spring water through a column was measured.

## Introduction

One of the serious problems associated with the utilisation of geothermal brine is the scale formation from inorganic salts, such as CaCO_3_, silica or CaSO_4_. Changes in pressure and temperature disturb the solution equilibrium and induce the formation of scale on the surface of production and reinjection wells, brine pipelines, valves, turbines and vapour–brine separators^1^. Such scale formation results in the gradual decrease of the brine flow rate and of the efficiency of heat-exchangers. In turn, these modifications result in exorbitant costs associated with equipment maintenance, replacement of unrecoverable parts and scale removal. The overall cost of scaling in the industrialised world is estimated to be 26,850 million USD^2,3^.

Various methods have been reported for the prevention of CaCO_3_ scale formation, such as use of pH modifiers, the addition of chemical inhibitors, including polyelectrolytes, metal ions, ethylenediaminetetraacetate, organophosphates or nanoparticles, and approaches involving the modification of the surface of the equipment^2–9^. Among these techniques, the addition of chemical inhibitors is the one most commonly utilised, due to the ease of its implementation. However, this approach necessitates the continuous addition of chemicals to a large amount of brine, which is expensive to do. In addition, there is concern about the environmental consequences of this approach, in particular with respect to groundwater and river water, of releasing, after completion of the heat utilisation process, large amounts of brine containing the chemical inhibitor, which may result in organic pollution and acidification.

Use of an electromagnetic field is another technique to prevent scale formation. This approach is based on the generation of electromagnetic fields within water conduits^10–12^. With respect to the technique based on chemical inhibitors, this method has the advantages of low cost and not requiring large amounts of chemicals, injection equipment and a chemical tank. Moreover, it does not cause pollution of the water environment. This has been used for more than a century, and a number of patents for magnetic scale treatment devices have been issued. However, the mechanisms and effects of electromagnetic fields as they relate to scale deposition prevention have not been identified.

Umeki *et al*. have investigated the effect of applying a weak alternating current electromagnetic field (magnetic flux density: ~150 µT) on the zeta potential of CaCO_3_ particles in water^13,14^. The zeta potential of CaCO_3_ particles dispersed in water changed as a result of the application of an electromagnetic field, and the magnitude of the change depended on the frequency of the electromagnetic field. When an electromagnetic field of 1–5 kHz frequency was applied, the zeta potential of CaCO_3_ particles was positive. When the frequency ranged between 6 and 10 kHz, the zeta potential changed from positive to negative. They suggested that one of reasons that electromagnetic fields inhibit scale formation has to do with the electrostatic repulsion between the negatively charged surface of CaCO_3_ particles and the negatively charged surface of vessels or pipes.

Several techniques have been used to evaluate scale formation, such as a direct visual inspection, weighing and monitoring the water flow rate through a column^15–18^. However, these methods to detect changes in the amount of deposited scale are time-consuming, taking a few days to months to be performed. Previously, we proposed the use of a fibre optic sensor that relies on an exposed fibre core, halogen light and a spectrometer to monitor scale formation in geothermal brines^19–22^. The ability of the fibre optic sensor to detect the presence of scale was based on the percentage of total internal reflection within the fibre optic core, which is influenced by the high refractive index of the scale formed on the surface of an exposed fibre core. The advantages of using this type of sensor include high sensitivity, real-time remote monitoring, heat resistance, small size, ease of handling and cost-effectiveness. This analytical method is suitable for use in a geothermal field to monitor scale formation and to determine the inhibition of scale formation within just a few hours.

In this study, field tests were conducted on water from a hot spring in Matsushiro, Nagano, Japan. The objective was to rapidly determine the effectiveness of electromagnetic treatment as a way to inhibit CaCO_3_ scale formation by the fibre optic sensor. The results obtained employing the fibre optic sensor were compared with the scale mass change measured by applying the quartz crystal microbalance (QCM) method. A typical column test was then conducted to verify the effect of the treatment on the flow properties of the water; in particular, the flow rate of hot spring water subjected to electromagnetic treatment was measured as it passed through a column packed with zirconia beads.

To the best of our knowledge, this report is the first published evaluation of the effectiveness of electromagnetic treatment for scale formation inhibition by rapid, real-time sensing carried out in a geothermal field. Notably, the results were carefully confirmed by comparison with those from QCM measurements and column flow tests. This study contributes to the understanding the mechanisms and effects of electromagnetic fields as they relate to scale deposition prevention.

## Results

### Sensor field test in 2015

**Figure 1:**
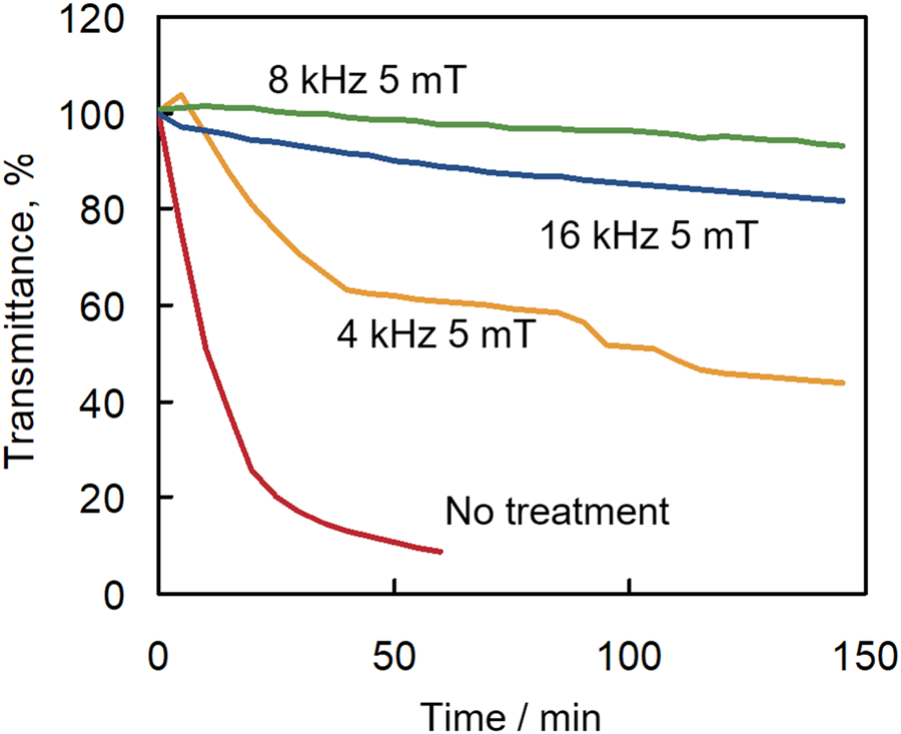
Transmittance response monitored using a fibre optic sensor at 1300 nm as a function of time after sensor immersion in hot spring water subjected (4 kHz 5 mT, 8 kHz 5 mT and 16 kHz 5 mT) or not subjected (No treatment) to treatment with an electromagnetic field. Tests were performed in 2015 on water samples from the hot spring of Matsushiro, Nagano.

A field test was performed in the hot spring in Matsushiro, Nagano, Japan on water characterised by high concentrations of calcium, carbonate, iron, sodium and chloride ions (Table 1). Figure 1 shows the transmittance responses at 1300 nm of a fibre optic sensor after the sensor was immersed in hot spring water subjected or not subjected to electromagnetic field treatment. The transmittance change was recorded in the 400–1600 nm range using a spectroscopic detector. Previously, we reported that the sensitivity of the fibre optic sensor for scale formation depended on the detection wavelength^19^. However, the relationship between these transmittance changes was the same at any detection wavelength employed in the field tests. Therefore, the wavelength of 1300 nm were selected in this study.

**Table 1:** Chemical composition of hot spring water samples collected in Matsushiro, Nagano, Japan in different years.

	14/11/2013^19^	17/11/2015	9/11/2016
Temperature (°C)	44.0	48.2	48.5
pH	6.65	6.62	6.80
Na (mg/L)	4,500	4,500	4600
K (mg/L)	480	530	510
Ca (mg/L)	1,100	1,000	760
Mg (mg/L)	310	300	300
Cl (mg/L)	8,600	8,700	8700
SO_4_ (mg/L)	200	200	210
HCO_3_ (mg/L)	2,300	2,300	1500
SiO_2_ (mg/L)	170	150	170
Fe (mg/L)	19	18	16

As can be evinced from the results reported in Fig. 1, under conditions of no applied electromagnetic field, the transmittance of the fibre optic sensor started to decrease immediately, and reached a value that was about 10% of the initial transmittance within 50 min, as a consequence of the deposition of scale on the surface of the fibre optic core. When an electromagnetic field was applied, the rate and degree of decrease in transmittance were smaller than the corresponding parameters measured in the ‘no-electromagnetic-field’ case. These results show that hot spring water treatment with an electromagnetic field effectively inhibited CaCO_3_ scale formation. In addition, the effectiveness of this approach to the inhibition of scale formation was successfully monitored with the fibre optic sensor.

### QCM test in 2015

**Figure 2:**
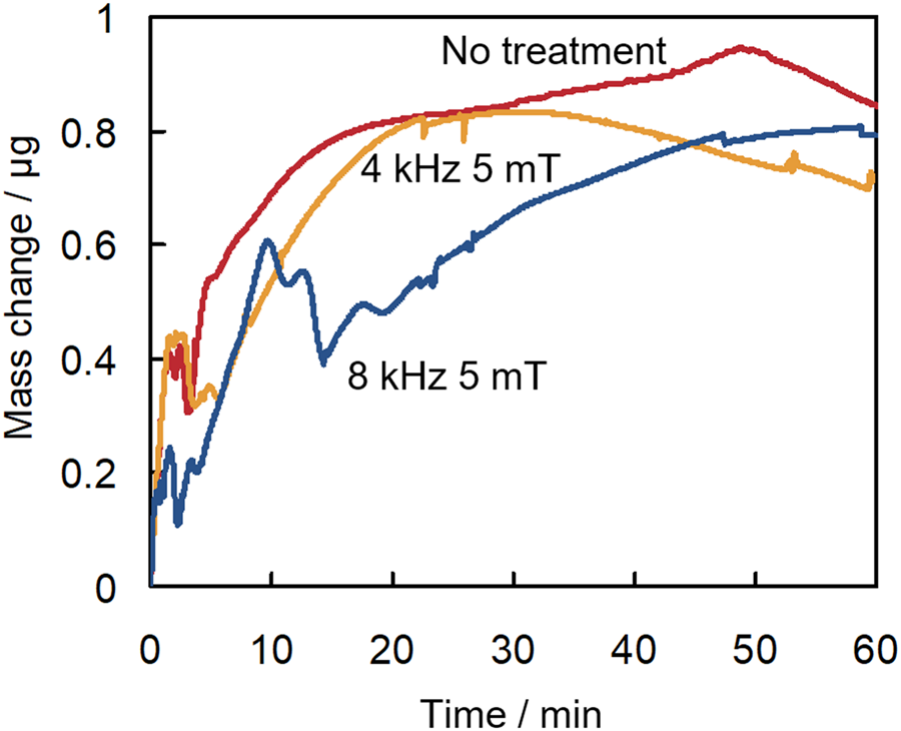
Mass change of the scale deposited on a quartz cell placed in hot spring water subjected (4 kHz 5 mT and 8 kHz 5 mT) or not subjected (No treatment) to electromagnetic field treatment as monitored by the quartz crystal microbalance analyser. Tests were conducted in 2015 on water samples from the hot spring in Matsushiro, Nagano.

QCM tests allow for the measurement of the mass change per unit area by detecting the change in frequency of a quartz crystal resonator resulting from the piezoelectric effect. QCM can detect mass changes at ng level. In the field test in 2015, the effectiveness of the electromagnetic treatment was also determined by QCM, which allowed the mass of the scale formed in hot spring water to be quantified. In particular, the increase in the mass of the scale deposited on the quartz crystal placed in the hot spring water sampled into a QCM cell could be measured directly. Figure 2 reports the change in mass of the scale formed on a quartz crystal surface in hot spring water either subjected or not subjected to electromagnetic treatment. The differences in mass change between treated and untreated water samples observed by QCM were smaller than those measured using the fibre optic sensor. However, a trend was apparent in the results of both sets of experiments, whereby electromagnetic treatment inhibited scale formation. Notably, water treatment with an electromagnetic field of 8 kHz frequency was observed to be as effective in inhibiting scale formation when measured by QCM as when it was done by fibre optic sensing.

### Column flow test in 2015

**Figure 3:**
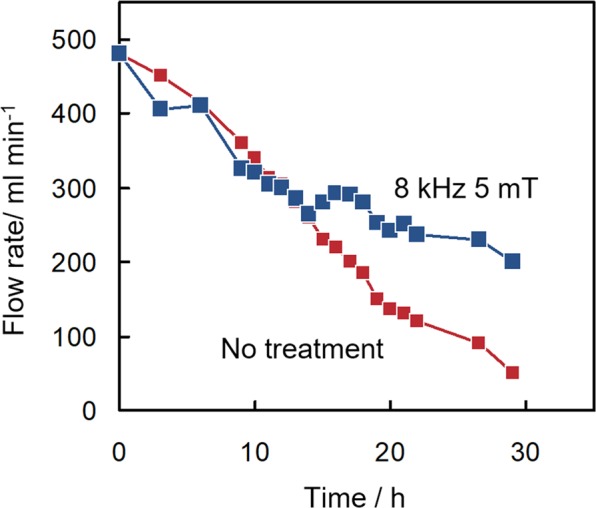
Flow rate of hot spring water subjected (8 kHz 5 mT) or not subjected (No treatment) to electromagnetic treatment through the column. These experiments were carried in 2015 on water samples from the hot spring in Matsushiro, Nagano.

Column flow tests enable researchers to directly measure the effect that scale formation has on the flow properties of a water sample. In fact, by performing these tests, the continuous monitoring of the flow rate, and hence the extent of scale formation in a column, can be achieved. Column flow tests were thus carried out to measure the effectiveness of the inhibition of scale formation achieved by the electromagnetic treatment of water samples. The values thus obtained were then compared with their counterparts measured via the fibre optic sensor test. Hot spring water samples subjected or not subjected to treatment with an electromagnetic field at 8 kHz frequency and 5 mT magnetic flux density flowed into the column. The flow rate of the hot spring water flowing out from the drain port was measured. The time-dependent changes in the flow rate of water samples subjected or not subjected to electromagnetic treatment are reported in Fig. 3. The flow rate decreased as a consequence of the formation of carbonate scale within the column. The effect on the flow rate of the treatment with an electromagnetic field of 8 kHz frequency and 5 mT magnetic flux density was clearly visible after 13 h.

### Field test in 2016

**Figure 4:**
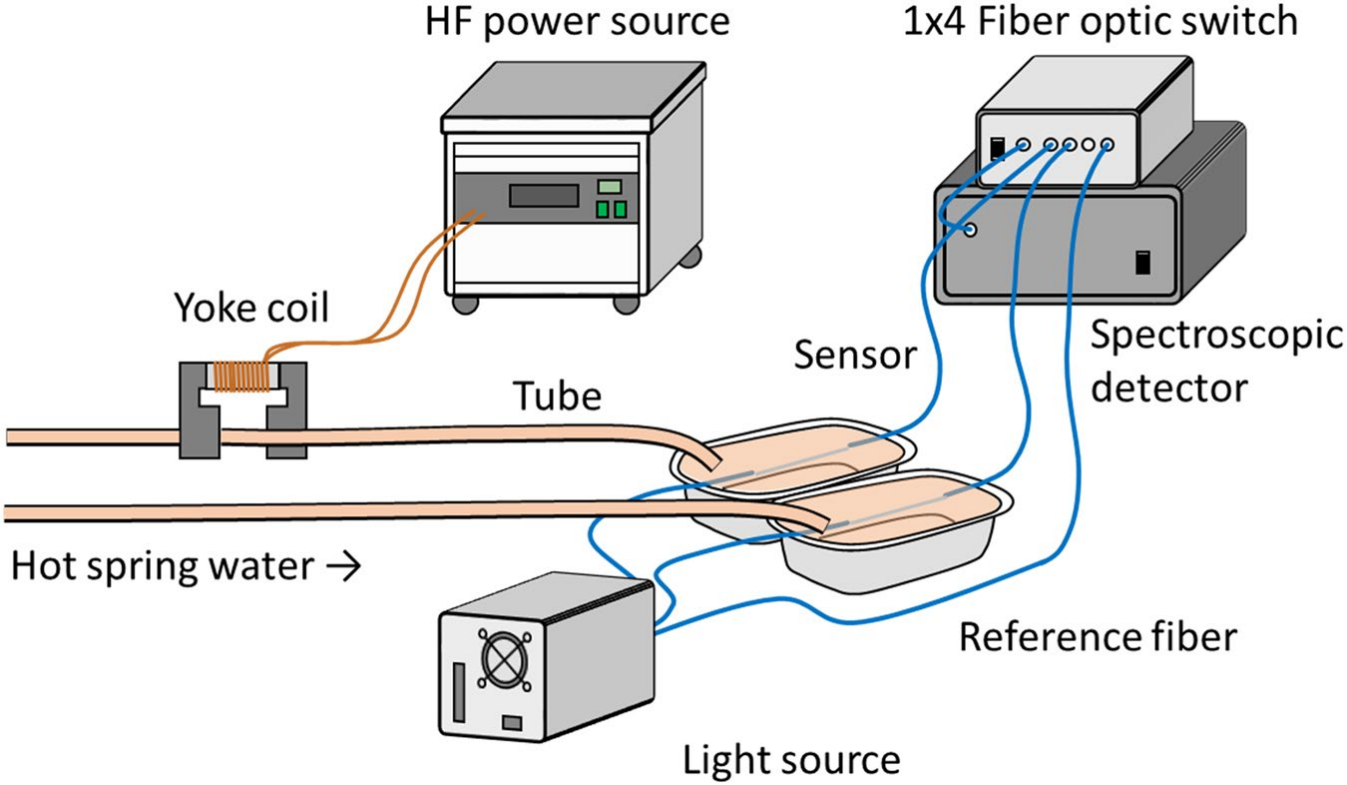
Schematic representation of the experimental setup utilised for the 2016 field test. Two tubes where geothermal water subjected and not subjected to electromagnetic treatment, respectively, flowed were located far enough from each other.

**Figure 5:**
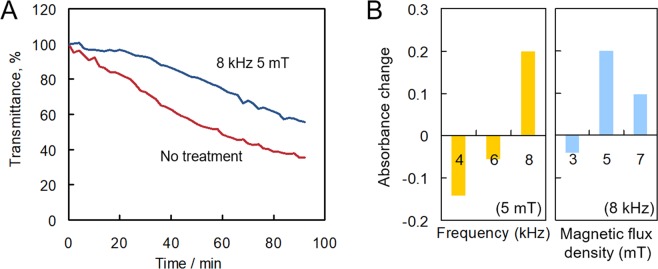
(**A**) Transmittance response measured using a fibre optic sensor at 1300 nm as a function of time after sensor immersion in hot spring water subjected (8 kHz 5 mT) or not subjected (No treatment) to electromagnetic field treatment. (**B**) Absorbance changes as a function of an electromagnetic field frequency and magnetic flux density, respectively, of hot spring water samples subjected to electromagnetic field treatment with respect to the case where water samples were not subjected to any electromagnetic field treatment; such changes were measured with a fibre optic sensor 80 min after electromagnetic field treatment. The relevant experiments were carried out in 2016 on water samples from the hot spring in Matsushiro, Nagano.

**Figure 6:**
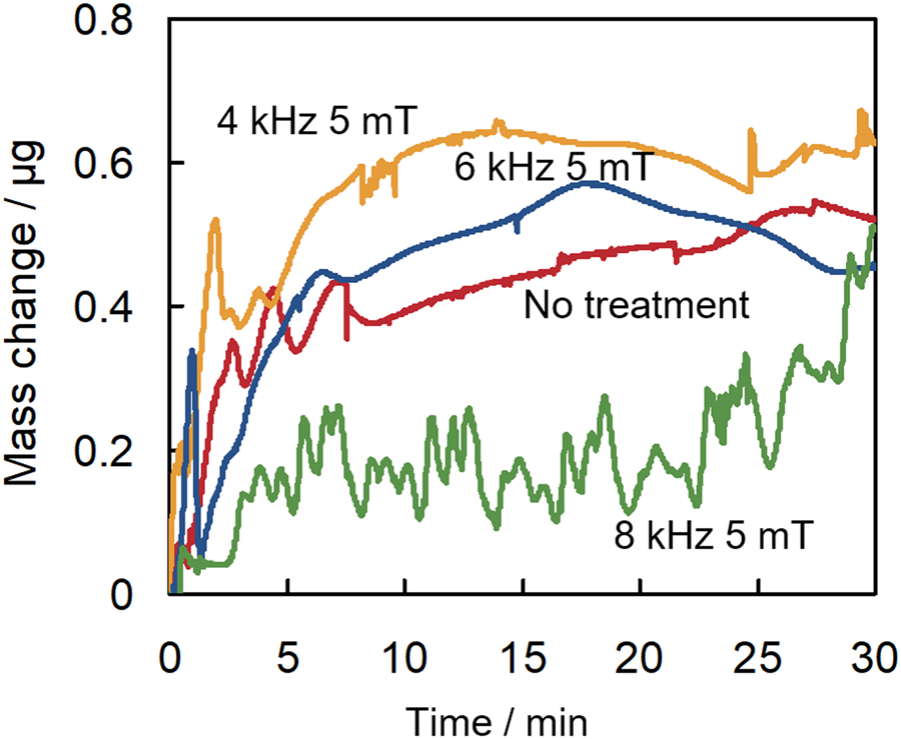
Mass change as monitored by the quartz crystal microbalance analyser method of scale deposited from hot spring water subjected (4 Hz 5 mT, 6 Hz 5 mT and 8 Hz 5 mT) or not subjected (No treatment) to electromagnetic field treatment. The relevant experiments were carried out in 2016 on water samples from the hot spring in Matsushiro, Nagano.

A field test was conducted again in 2016 in Matsushiro to verify the effect that water electromagnetic field treatment had on scale formation. In the experiments in 2016, the transmittance change associated with scale formation varied greatly in measurements performed on water samples not subjected to electromagnetic treatment. In 2016, the concentrations of Ca^2+^ and HCO_3_^−^ were lower than observed values in 2013 and 2015 (Table 1). In addition, Ca^2+^ concentration varied during the time in which the field test was conducted in 2016. The hot spring water in Matsushiro sources from a mixture of fossil sea water and rain water. It is suggested that the hot spring water in 2016 had been highly diluted by rainwater compared to other years. Therefore, evidence seems to suggest that the instability of the concentrations of chemicals in the water of the hot spring affected the measurements performed with the sensor. Consequently, an optical switch was connected to the fibre optic sensor to achieve the multipoint monitoring depicted in Fig. 4. In this system, the transmittance responses of water samples subjected and not subjected to electromagnetic treatment could be simultaneously monitored in the same experimental conditions. Figure 5 shows the transmittance responses to scale formation measured in hot spring water samples subjected and not subjected to treatment with an electromagnetic field of 8 kHz frequency and 5 mT magnetic flux density. The decrease in transmittance observed in water samples not subjected to electromagnetic treatment was smaller than the corresponding parameter measured in 2015. This observation may be due to the decrease in the concentrations of Ca^2+^ and HCO_3_^−^ in the hot spring water alluded to above. Although the inhibition of scale formation was indeed observed under an applied electromagnetic field of 8 kHz frequency and 5 mT magnetic flux density, the observed effect was smaller than that observed in 2015. The absorbance changes between water samples not subjected and those subjected to electromagnetic field treatment are shown in Fig. 5(B). Previously, we reported that the change in absorbance measured by the fibre optic sensor was related to the change in mass of the scale deposited on the fibre core^22^. When the frequencies of the applied electromagnetic fields had values of 4 and 6 kHz, the rate of scale formation was enhanced in relation to the case where the frequency of the applied electromagnetic field was 8 kHz. This trend was similar to that identified based on results from the QCM test in 2016 (Fig. 6). With respect to varying magnetic flux densities, electromagnetic fields of 8 kHz frequency and 5 and 7 mT magnetic flux densities demonstrated a tendency to inhibit scale formation; however, this tendency was not observed when an 8 kHz electromagnetic field of 3 mT magnetic flux density was utilised.

## Discussion

Based on the results of the sensor field test performed in 2015, the change in transmittance varied depending on the frequency of the electromagnetic field applied to the hot spring water. As mentioned above, the zeta potential of CaCO_3_ particles present in water depends on the frequency of the electromagnetic field applied to the water sample. In previous laboratory experiments, the zeta potential of CaCO_3_ particles changed from positive to negative as a consequence of the application of electromagnetic fields with frequencies above 6 kHz^13,14^. In the field test, the zeta potential of CaCO_3_ scale formed in the hot spring water might similarly change from positive to negative when an electromagnetic field is applied. The inhibition of CaCO_3_ scale formation resulting from electromagnetic field treatment could be due to electrostatic repulsion between the negatively charged surface of CaCO_3_ scale particles and the negatively charged quartz fibre core of the sensor.

In the QCM test, a similar tendency, whereby the electromagnetic treatment of water inhibited scale formation, was observed. However, the differences in extent of scale formation between water samples subjected to electromagnetic field treatment and those not subjected to this treatment were small compared to their counterparts measured in the sensor field test. In QCM experiments, the mass of materials deposited on the crystal surface at the bottom of the cell was measured. In the case of field test, the QCM measured not only mass of the scale directly formed on the crystal surface, but also that deposited on the crystal surface after being precipitated in the hot spring water. Calculations made on the basis of Stokes’ law indicate that calcium carbonate particles with weights in the ng–µg range have sufficient settling velocity to be deposited at the bottom of the cell in the timescale of this experiment. As gravity will strongly influence the mass change of particles as compared to electromagnetic field, the differences in effect of electromagnetic treatment monitored by QCM was not clear compared to the sensor operated in flow system. From the result, the effect in electromagnetic treatment can be detected slightly as direct mass change by QCM. Moreover, the fibre optic scale sensor that can be used in flow systems was more suitable to the evaluation of the effectiveness of electromagnetic treatment for scale formation in hot spring water.

In the column flow test in 2015, the effect of the treatment of water with an electromagnetic field was clearly observed. Monitoring the water flow rate gives direct information on the amount of scale deposited on the column surface. We have carried out column flow tests in various geothermal fields, including that in Matsushiro^17,23–27^. Our results indicate that treatment of water with an electromagnetic field is an effective approach to inhibiting scale formation. In addition, the effectiveness of this approach can be easily measured using a fibre optic sensor via a protocol that can be completed in 1/30 of the time needed to perform the column test.

In experiments conducted as part of the sensor test performed in 2016, treating water samples with electromagnetic fields characterised by frequencies of 4 and 6 kHz slightly enhanced the extent of scale formation with respect to the case where no-electromagnetic-field had been applied. Furthermore, the effects of treatment with an 8 kHz electromagnetic field seemed to be smaller than those measured in 2015. In laboratory experiments performed previously, the zeta potential of CaCO_3_ particles increased from ~+8 mV to ~+20 mV as a consequence of the application of an electromagnetic field with progressively increasing frequency, from 0 kHz (no applied field) to 4 kHz. Between 4 kHz and 10 kHz, the zeta potential of CaCO_3_ particles decreased, and, between 5 and 6 kHz, the sign of the zeta potential changed from positive (+6 mV at 5 kHz) to negative (−5 mV at 6 kHz). This trend, whereby the value of the zeta potential of CaCO_3_ particles decreases, after initially increasing, as the frequency of the applied electromagnetic field increases, matches the results obtained in the experiments performed using the fibre optic sensor in 2016. Therefore, it is suggested that the electromagnetic field frequency at which the zeta potential on the surface of CaCO_3_ particles assumes a zero value has changed between the field test performed in 2015 and that performed in 2016. When, in experiments conducted in 2016, the frequency of the electromagnetic field was 4 kHz, scale formation was accelerated with respect to the no-electromagnetic-treatment scenario, due to an increase in the (positive) value of the zeta potential of scale particles. When the frequency was 6 kHz, the zeta potential of the scale particles decreased slightly, leading to a situation whereby the rate of scale formation was close to that observed when no-electromagnetic-field treatment had been applied. In the 8 kHz frequency case, the sign of the zeta potential of scale particles had turned to negative, leading to an inhibition of scale deposition. Hence, the frequency of the electromagnetic field applied to hot spring water may be insufficient for the zeta potential of the scale particle to be the lowest in the field test 2016, as compared with the result in 2015. The result according to which scale formation was inhibited by water treatment with an electromagnetic field characterised by a frequency of 8 kHz and accelerated by treatment with fields characterised by 4 kHz or 6 kHz frequencies was certainly similar to that obtained in the QCM field test performed in 2016. However, the reasons for the shift observed between 2015 and 2016 in the frequency at which the zeta potential of scale particles assumes a value of zero remain unclear. One contributing factor (if not the main reason) is likely to be a change in the quality of the water from the hot spring. The effects that the calcium carbonate saturation index in the hot spring water and the frequency of the applied electromagnetic field have on the zeta potential of precipitated CaCO_3_ particles could be investigated in future studies. If the quality of geothermal water influenced the optimal conditions for water treatment with an electromagnetic field, it is important to evaluate easily and simply the scale formation by the fibre sensor capable of simultaneous multipoint analysis in geothermal fields.

Magnetic flux density is related to the intensity of the magnetic field. Based on the results of the present study, a magnetic flux density of 3 mT for an electromagnetic field of 8 kHz frequency was not high enough for the inhibition of scale formation to be observed in hot spring water. When this parameter assumed values of 5 mT and 7 mT, on the other hand, scale formation inhibition was indeed observed. However, the absorbance observed with an electromagnetic field with a magnetic flux density of 7 mT was slightly smaller than that observed with an electromagnetic field with a magnetic flux density of 5 mT. This effect should be studied under conditions whereby the frequency of the applied electromagnetic field is higher than 8 kHz, so as to maximise scale formation inhibition. In short, the effectiveness of the electromagnetic treatment for scale formation inhibition was affected by the values of the magnetic flux density.

Overall, the effectiveness of the electromagnetic filed treatment of hot spring water at Matsushiro for scale formation inhibition was successfully monitored with the fibre optic sensor. The effectiveness of this treatment was influenced by the values of the frequency and the magnetic flux density. However, these values should not be apply for other geothermal brine or hot spring water because the effectiveness of the electromagnetic filed treatment will be affected by the quality of the water. The optimal conditions with respect to frequency and magnetic flux density should be carefully evaluated in geothermal fields via the fibre optic sensor approach.

## Methods

### Materials

A step-index multimode optical fibre (FT200EMT; Thorlabs, USA) with a 200-μm-diameter fused silica core was used. The fibre core has a refractive index of 1.451, and it is surrounded by a TEQS^TM^ polymer cladding with a refractive index of 1.392 at 1020 nm. The sensor was fabricated by removing the 16-cm-long cladding from the centre of the fibre. The sensor was connected to a white-light source (ELI-050J-OPT3077; Mitsubishi Rayon, Japan) and an SA-100VRD visible–near-infrared (Vis–NIR) spectroscopy detector (Lambda Vision, Japan). Sensor transmittance values were acquired by recording the light intensity through the fibre before the said fibre was exposed to conditions whereby scale would form on its surface (*I*_0_) and the light intensity after fibre exposure to scale formation (*I*). Sensor transmittance in the fibre sensor was defined as T (%) = (*I*/*I*_0_) × 100.

A yoke coil for generating electromagnetic fields in the high-frequency range was prepared with a U-shaped Mn/Zn ferrite with a 1 mm diameter formal wire wound 24 times (*L* = 124.5 µH, *Z* = 6.2598 Ω (at 8 kHz)). The two ends of the wire were connected to a high-frequency power supply.

For QCM measurements, a quartz crystal with a gold layer of 100 Å thickness on a titanium adhesive layer (BAS, Japan) was used. A Teflon cell attached the quartz crystal was used in a thermostatic Faraday case (TB-1; BAS, Japan). A model ALS401C EQCM analyser was used to perform QCM measurements.

### Field study in 2015

A field study was carried out from November 16^th^ to November 20^th^, 2015 using water from a hot spring in Matsushiro, Nagano, Japan. The chemical composition of the hot spring water is summarised in Table 1. For the fibre optic sensor study, hot spring water was made to flow over the fibre optic sensor fixed inside an 11-litre bucket with tension applied to both ends. The coil was attached to the tube that directed hot spring water to the sensor.

In the QCM tests, hot spring water treated with an electromagnetic field was filtered through a membrane filter (pores: 0.22 µm), and 4 ml of the filtered water was transferred to a Teflon cell with a syringe. The mass change of the scale deposited on the quartz crystal in the cell was monitored using an EQCM analyser.

The column test was carried out according to the protocol described in previous studies from our group^17,23–27^. The test cell in a stainless-steel column contained Teflon cells inside (30 mm inner diameter × 500 mm length, 282 mL internal volume). The cell was filled with zirconia beads (1 mm in diameter). Hot spring water treated with electromagnetic field flowed into the column. The flow rates (L/min) of hot spring water flowing out from the drain port were measured.

### Field study in 2016

The second field test was conducted between November 8^th^ and November 11^th^, 2016. In the experiments conducted as part of this test, a 1 × 4 optical switch was connected to the fibre optic sensor, to realise the multipoint monitoring depicted in Fig. 4. Two input ports at the optical switch were used for the sensors in conditions whereby water had been subjected or not subjected to electromagnetic treatment. The unprocessed fibre was connected to another port and is used as a reference for optical intensity. An output port was connected to the spectroscopic detector with a FT200EMT fibre. A computer controlled the switch and the detector measured the intensity of the light passing through the fibres. Two tubes where hot spring water subjected and not subjected to electromagnetic field treatment, respectively, flowed were located far enough apart to avoid ‘no-electromagnetic-field’ water being influenced. The QCM test was carried out implementing the same protocol utilised for the 2015 test.
